# Case of Tetanus—Recovery

**Published:** 1864-01

**Authors:** W. A. Davis

**Affiliations:** Chimborazo Hospital.


ART. IV.—*Case of Tetanus—Recovery.* Reported by Surg. W. A. DAVIS, Chimborazo Hospital.

Serg't M. J. Dunkum, company "D," 21st Va. infantry, admitted in Chimborazo Hospital, No. 4, Nov. 10th; was wounded on the 26th October by a cannon ball, and on 27th October right thigh was amputated, lower part of upper third by a circular operation. Symptoms of tetanus appeared about 6th inst. When admitted the tetanus was marked; lower maxilla fixid, but no tetanic convulsions; able to take nothing by the mouth except fluids; posterior muscles of neck also rigid, and painful twitching of stump; profuse perspiration; pulse 110; some appetite, and bowels regular. Stump in an unhealthy condition; a large slough, some three inches deep, separating on the external side, the line of demarkation being very plain; bone protruding about an inch, and a fungus growth from the medullary canal.

*Treatment.*—Cleaned the wound, and drew it together by adhesive plaster as well as possible; milk-punch, soup, eggs, &c., ordered; cit. iron and quinine and morphine, to procure sleep and rest (grains ii. to iii. daily); Labaraque's disinfectant used.

Nov. 15th.—General symptoms rather better; perspiration continues; sleeps well, and eats moderately.

The sloughing mass has dropped off, and the stump looks better and granulating.

Nov. 20th.—Tetanus slowly passing off; good appetite; sleeps well after taking i. grain morphine at night; stump doing well.

Nov. 26th.—Improving in every way.

